# Supplementary material for: Netcombin: An algorithm for constructing optimal phylogenetic network from rooted triplets
Source: PLoS One. 2020 Sep 18;15(9):e0227842. doi: 10.1371/journal.pone.0227842 (PMC7500971; doi:10.1371/journal.pone.0227842)
Supplement: S4 File — (ZIP) [file pone.0227842.s004.zip › SequenceData-Triplet/sequenceData-10-20-30-0-Triplet/length 200, numseq 30, rate 10^-7/9 seq.pdf]

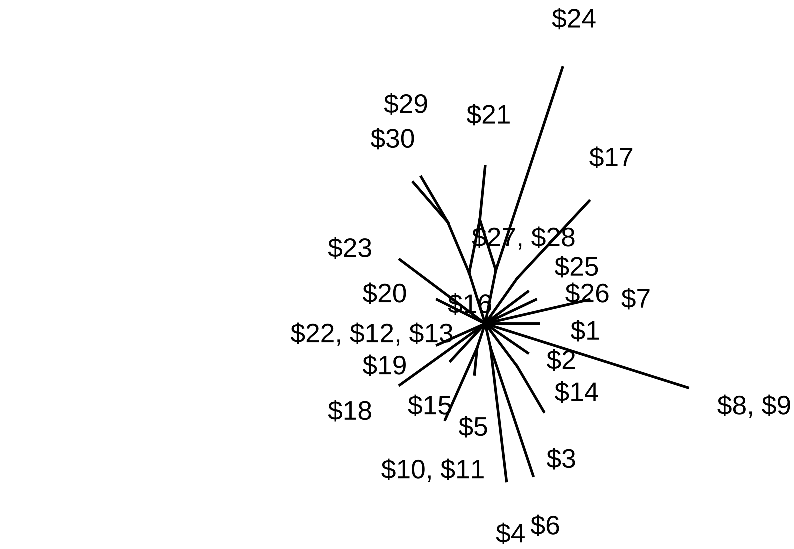
0.0010
$24
$29
$21
$30
$17
$27, $28
$23
$25
$20
$26
$7
$16
$22, $12, $13
$1
$19
$2
$14
$18
$15
$8, $9
$5
$3
$10, $11
$4
$6
